# International Cross-Sectional Study on the Effectiveness of Okada Purifying Therapy, a Biofield Therapy, for the Relief of Various Symptoms

**DOI:** 10.1089/acm.2019.0264

**Published:** 2020-07-30

**Authors:** Kiyoshi Suzuki, Seiya Uchida, Tomoaki Kimura, Hideaki Tanaka, Hiroshi Katamura

**Affiliations:** ^1^ General Incorporated Foundation MOA Health Science Foundation, Tokyo, Japan.; ^2^ Tokyo Ryo-in MOA Takanawa Clinic, Tokyo, Japan.

**Keywords:** biofield therapy, energy healing, international study, ethnicity, effectiveness, adverse events

## Abstract

***Objective:*** To investigate whether differences exist in the effectiveness/safety of a single session of Okada Purifying Therapy (OPT), a type of biofield therapy, among those from different ethnicity/cultures, and to analyze factors associated with the outcomes in a real-world setting.

***Design:*** Pre–post test design using convenience sampling methods.

***Setting:*** Home setting.

***Subjects:*** A total of 11,303 individuals aged 16 years or older from 14 different countries (>1000 individuals each from Japan, the United States, Thailand, Chile/Peru, and <200 individuals each from Portugal, Spain, Argentina, Mexico, Brazil, South Korea, Taiwan, Belgium, and France). More than 50% of the subjects were themselves OPT practitioners, and more than 50% of the treatments were administered in an environment where the practice of OPT was promoted.

***Intervention:*** Participants received a single session of OPT lasting 30 min or longer from the volunteer practitioners. They self-reported the changes in overall symptoms, physical pain, anxiety/depression, and dizziness/palpitation.

***Outcome measures:*** Improvement/exacerbation rates of each symptom and factors associated with symptom improvement were analyzed.

***Results:*** Of the participants, 77.5%, 75.6%, 78.4%, and 73.8% reported an improvement of overall symptoms, physical pain, anxiety/depression, and dizziness/palpitation, respectively. The improvement rates were consistently higher among participants from Chile/Peru than those from Japan, the United States, and Thailand (*p* < 0.001), and among those who had received a longer therapy (*p* < 0.001). Spanish/Portuguese speaking countries almost always showed high improvement rates; conversely, Japan showed a lower rate in each symptom. Participants' gender, reasons for participation, previous experience, and location of the session were also associated with the improvement of different symptoms. These findings occurred regardless of the participants' age or presence/absence of illness. In terms of safety, the exacerbation rates of symptoms were 2.8%, 2.5%, 0.8%, and 1.7%, respectively. Of those who expressed symptoms exacerbation, 71.6% recovered in a few hours. None of them needed emergency medical treatment.

***Conclusions:*** In those who were often sympathetic to OPT and/or in an authorized location, OPT was effective and safe across countries with ethnic/cultural differences; however, participants' country of residence and duration of the session were independently associated with the changes in various symptoms. (ClinicalTrials.gov NCT03994809).

## Introduction

Biofields are explained by Jain et al. as “endogenously generated fields, which may play a significant role in information transfer processes that contribute to an individual's state of mental, emotional, physical, and spiritual well-being.”^1^ They further describe biofield therapy as “noninvasive, practitioner-mediated therapies that explicitly work with the biofield of both the practitioner and recipient to stimulate a healing response in the recipient.”^1^ Healing Touch, Reiki, Therapeutic Touch, and Johrei are examples of such practices.^1^

The scientific mechanism of biofield therapy (or energy healing) remains to be clarified^1–5^; meanwhile, the therapy has been reported to ameliorate pain in different illnesses,^1,6–12^ psychologic symptoms, and/or anxiety.^3,8,10–14^ Biofield therapy also reportedly influenced the autonomic activity in patients early after acute coronary syndrome^15^ and increased in positive emotional state and well-being.^3,11,15,16^ In the context described by Jain et al.,^1^ Okada Purifying Therapy (OPT),^17,18^ initially formulated by Mokichi Okada (1882–1955) in the mid 1930s in Japan, is a type of biofield therapy.

## Okada Purifying Therapy

OPT has been practiced generally in home settings to relieve various symptoms. The general principles underlying OPT are based on Okada's concepts.^17,18^ He stated that all physical materials and creatures are the mixture of the “body” in the present world and the “spirit” belonging to the spiritual world, which mutually interact. Synthetic substances or metabolic wastes in the body become toxins, which cause many forms of illnesses, and transform into defilement in the spirit. Furthermore, bad behavior or idea produces defilement in the spirit and become toxins in the body. Removal of toxins in the body, that is, elimination of defilement in the spirit, results in the fundamental solution of any type of illness and leads to true happiness.

During the training course, OPT practitioners study the concept of OPT, the reason of its effectiveness, and methods to remove toxins from the body. Stiff and/or warm spots on the body represent accumulated toxins, which are the key areas to administer OPT. With permission from the recipient, the practitioner places his/her palm on the recipient's head, neck, shoulder, upper back, and lower back in sequence to search for stiff and/or warm spots emanating from the body.

During the therapy, the practitioner absorbs a “life-force energy” permeating in the universe and raises his/her hand forward toward the recipient with the palm directed toward the recipient, with feelings of devotion toward symptom improvement and happiness for the recipient. The practitioner radiates the “life-force energy” from his/her palm toward the key areas on the recipient's body, envisioning the energy penetrating through the recipient's body. The practitioner uses his/her hands alternately during the administration of OPT. The distance between the palm and the body is usually 1–2 feet, with each session typically lasting 30 to 60 min.

According to Okada, OPT invigorates the self-healing ability to remove accumulated toxins, thereby facilitating physical-mental-spiritual health for both recipients and practitioners.^17,18^ The practitioner explains the concept of OPT described above to the recipient before the treatment.

Beginning in 2000, MOA International Corporation (MOA) developed an accreditation system for OPT.^17^ Anyone can practice OPT as noncertified practitioners by taking a basic training course. To be certified as practitioners, individuals can take an advanced course and pass an examination approved by the corporation. More than 50,000 out of ∼800,000 practitioners in Japan and more than 20,000 out of 200,000 practitioners outside Japan had been certified as of February 2020.

The research team previously reported that the power values of alpha waves detected by electroencephalograms increased^19,20^ and the heart rate variability in electrocardiograms changed in OPT sessions, during which the recipients were unaware of whether or not they were receiving the therapy.^19,21,22^ In a separate cross-sectional study in Japan, ∼70% of the participants reported improved symptoms after a single OPT session, although the improvement rates varied according to gender, location and duration of the session, and reasons for using OPT.^23,24^ Long-term practice of OPT has been described to improve menopausal symptoms,^25^ anemia, and survival rate in patients with sickle cell disease,^26^ and widespread pain in those with fibromyalgia.^27^

To promote physical-mental-spiritual well-being, MOA recommends combination of health programs (Okada Health and Wellness Program), which consists of OPT along with diet and art components.^17^ The team reported that the regular practice of Okada Health and Wellness Program helped ∼80% of patients with hypertension to become normotensive, and ∼30% either reduced/stopped taking medication.^28^ Further, simultaneous practice of OPT, diet, and art components were more likely to improve quality of life (QOL)^29^ evaluated with the 10-item MOA quality of life questionnaire (MQL-10),^30^ which is described further in the Methods section.

While biofield therapy often includes a holistic and spiritual meaning, ethnic differences were reportedly observed in the use of energy therapies^31,32^ and spiritual practices.^33,34^ The outcomes of biofield therapy may be influenced by individual's ethnic/cultural background as well as their expectation, situations of receiving the therapy, and relationship with the practitioner.^35–38^ The degree of acculturation and/or duration of residence in a certain country may also play a role.^39^ Ghiasuddin et al. reported that pediatric oncology patients who received healing touch and their parents in Hawaii had attitudes/beliefs around health care that were rooted in their traditional cultural values with the younger generation being less traditional.^40^ To the extent of the research team's knowledge, it has not been reported whether the recipient's ethnic/cultural background influences the outcomes of biofield therapy. Therefore, the present study aimed (1) to investigate whether the effectiveness/safety of a single session of OPT in various settings differ across different ethnicities/cultures, and (2) to analyze factors associated with the outcomes in a real-world setting. This study was conducted in accordance with the Declaration of Helsinki 1975, as revised in 2013, and was approved by the Institutional Review Board and the Research Ethics Committee of the MOA Health Science Foundation.

## Methods

This study aimed to include as many participants as possible in a real-world setting, which would have been impaired by introducing artificially created control groups. Therefore, the research team chose a design to recruit the participants using convenience sampling methods without assigning control groups.

### Investigators

The first author delivered a lecture to OPT instructors, ∼100 in Japan and 25 outside Japan, to explain the purpose of the study and to provide guidance for administering the questionnaires. This initial lecture was video recorded, which the instructors used to train other certified OPT practitioners as investigators. The training was conducted several times each in 222 locations in Japan and 30 times in 15 locations outside Japan. Explanation was provided on how to recruit participants, to administer OPT, and to use record sheets.

The investigators were not financially reimbursed for conducting the OPT sessions or for participating in this study. Noncertified practitioners took part in this study as recipients, but not as investigators. The OPT sessions were carried out according to the protocols provided by MOA, the outline of which is described in the introduction.

### Participants

The research team developed a Japanese version of the information sheet and the questionnaires. They were translated into English, Spanish, French, Portuguese, Thai, Chinese, and Korean languages under the responsibility of each regional headquarter of MOA. The investigators used the information sheet and word-of-mouth to recruit participants in 14 different countries. From January 2008 to September 2010, subjects received a single session of OPT from the investigators at the MOA's affiliated institutes or workshops, the investigator's house, or the participant's home.

Inclusion criteria were as follows: (1) individuals who agreed to receive a single session of OPT for 30 min or longer from the investigators, (2) were able to self-evaluate the changes in their symptoms, (3) competent to answer the questionnaires, and (4) aged 16 or older. All individuals agreed to participate without receiving an honorarium. Certified practitioners could participate as both investigators and participants, provided that they met the inclusion criteria. There were no specific exclusion criteria for this study.

### Questionnaires

**FIG. 1.**
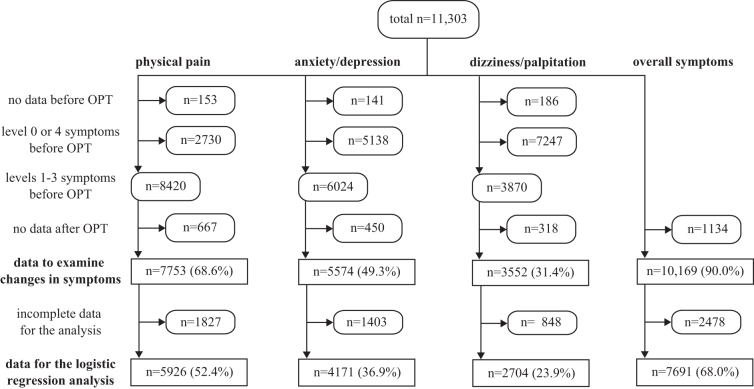
Number of analyzed data of each symptom. Individuals who met the inclusion criteria are shown as numbers and proportions (%) of the total participants. no data, the level of symptoms was not reported; incomplete data, one or more characteristics were not reported. OPT, Okada Purifying Therapy.

Participants completed a demographic questionnaire before the OPT session. They self-evaluated the severity of the following categorized symptoms by means of a 5-point Likert-type scale ranging from 0 (none) to 4 (extreme) before and after an OPT session: physical pain, anxiety/depression (psychologic symptoms), and dizziness/palpitation (autonomic symptoms). They also self-reported the changes in overall symptoms (improved, no change, or exacerbated) after the session (Fig. 1). Those who expressed symptom exacerbation were asked to report afterward what they had done to relieve their symptoms and when they recovered from the exacerbation.

To evaluate participants' QOL, a free QOL assessment questionnaire applicable to a large sample size was required. Therefore, the MQL-10 questionnaire^30^ was chosen for use, which was previously developed for this study. The MQL-10 score ranges from 0 to 40, with higher scores indicating better QOL, and its minimal important difference is three points. The Cronbach's alpha coefficient of the MQL-10 was 0.872 at baseline and 0.879 at follow-up, both above cutoff point of 0.8, suggesting a good internal consistency. The correlation coefficient with WHOQOL-26 and SF-36 (mental health domain) was 0.81 and 0.64, respectively.^30^ Participants completed the MQL-10 questionnaire before the OPT session.

### Analyses

The research team classified changes in symptoms for each category into “improved,” “no change,” or “exacerbated” by comparing the symptom levels before and after the OPT session. The team used the data from the participants who had symptom levels of 1, 2, or 3 at baseline (Fig. 1). This criterion was used because a “no change” response from participants who had no symptoms (level 0) at baseline would have a different meaning than a “no change” response would have for participants whose symptoms had existed before the OPT session. Similarly, participants with extreme symptoms (level 4) at baseline could not report that the treatment exacerbated their symptoms because level 4 was the highest level available, with the result that participants' reports of “no change” would not reflect an exacerbation of symptoms.

The authors used the Japanese SPSS version 25 (IBM SPSS Statistics, Tokyo, Japan) for data analyses. To account for potential risk of misclassification of participants from Chile being sorted into those from Peru, in the regional headquarter of both countries, data from Chile and Peru were classified into Chile/Peru group. Due to the limited number of participants from Belgium and France, data from each country were classified into Belgium/France group. More than 1000 individuals each from Japan, the United States, Thailand, and Chile/Peru, and 26–136 subjects each from the other nine countries participated and met the inclusion criteria of this study (Table 1).

**Table 1.** Basic Characteristics of the Participants (Total *n* = 11,303)

Country of residence (*n* = 11,303)	
Japan	7187 (63.6%)
The United States	1276 (11.3%)
Thailand	1176 (10.4%)
Chile/Peru	1068 (9.5%)
Portugal	136 (1.2%)
Spain	116 (1.0%)
Argentina	106 (0.9%)
Mexico	83 (0.7%)
Brazil	56 (0.5%)
South Korea	44 (0.4%)
Taiwan	29 (0.3%)
Belgium/France	26 (0.2%)
Age (*n* = 11,191)	
16–49	3304 (29.5%)
50–69	4557 (40.7%)
≥70	3330 (29.8%)
Gender (*n* = 11,041)	
Male	2492 (22.6%)
Female	8549 (77.4%)
Presence of illness (*n* = 10,498)	
Absent	5237 (49.9%)
Present	5261 (50.1%)
Reasons for participation (*n* = 10,575)	
Health promotion	3769 (35.6%)
Symptom relief	2327 (22.0%)
Suggestion of others	4479 (42.4%)
MQL-10 score (10,626)	
Low (≤25)	3489 (32.8%)
Moderate (26–30)	4007 (37.7%)
High (≥31)	3130 (29.5%)

Categorical values are shown as numbers and proportions (%).

MQL-10, 10-item MOA quality-of-life questionnaire.

Because of large discrepancies in the number of participants between countries, the following statistical analyses were conducted between the participants from Japan, the United States, Thailand, and Chile/Peru. A Kruskal–Wallis test was used to compare ordinal variables between four countries. If results indicated statistical significance (*p* < 0.05), the Mann–Whitney test was further conducted to compare two countries, in which a *p*-value of 0.0083 was designated as the cutoff point for statistical significance according to the Bonferroni correction.

Then the authors conducted the logistic regression analyses of the factors associated with the changes in symptoms (Fig. 1). The category of “no change or exacerbation in symptoms” was used as the reference. Before conducting analysis, the team created three subgroups for each variable to divide the total data-units into comparable sets. The age groups (16–49, 50–69, and ≥70 years), duration groups (30, 31–50, and ≥51 min), and MQL-10 groups (≤25, 26–30, and ≥31) included an approximately equal number of individuals in each subgroup. A *p*-value of less than 0.05 was considered statistically significant.

To compare the results of the research team's previous study from 2007, which was conducted within Japan,^24^ Japan's data in the present study were independently analyzed to determine the factors associated with the changes in physical pain, anxiety/depression, and dizziness/palpitation.

To estimate the influence of qualification as an OPT practitioner on the changes in symptoms, the improvement rates in various symptoms were compared between those with never/seldom OPT experience and those with qualification of OPT practitioner in participants from Japan, the United States, Thailand, and Chile/Peru, respectively. The Mann–Whitney test was used for the analyses, and a *p*-value of less than 0.05 was considered statistically significant.

## Results

A total of 11,303 individuals from 14 different countries met the inclusion criteria for the analyses. Among participants who reported each question items appropriately, 7187/11,303 (63.6%) were from Japan, 7887/11,191 (70.5%) were aged 50 years or older, 8549/11,041 (77.4%) were female, 5261/10,498 (50.1%) reported presenting illness, and 4479/10,575 (42.4%) participated through suggestion of others (Table 1). In terms of characteristics associated with OPT, 5607/11,114 (50.4%) were qualified OPT practitioners, 4849/11,023 (44.0%) experienced OPT for the first time or for the first time in a long time, 5480/10,846 (50.5%) received the OPT session at an MOA affiliated institute or workshop, and 4737/11,303 (41.9%) underwent intervention which lasted for 30 min (Table 2).

**Table 2.** Participants' Characteristics Associated with Okada Purifying Therapy (Total *n* = 11,303)

OPT practitioner (*n* = 11,114)	
No	5507 (49.6%)
Yes	5607 (50.4%)
Previous OPT experience (*n* = 11,023)	
Never/seldom	4849 (44.0%)
Sometimes/regularly	6174 (56.0%)
Location of OPT administration (*n* = 10,846)	
Participant's home	3071 (28.3%)
MOA's institute/workshop	5480 (50.5%)
Investigator's house	2295 (21.2%)
Duration of OPT administration (*n* = 11,303)	
30 min	4737 (41.9%)
31–50 min	3244 (28.7%)
≥51 min	3322 (29.4%)

Categorical values are shown as numbers and proportions (%).

MOA, MOA International Corporation; OPT, Okada Purifying Therapy.

In each country, female participants accounted for more than 65%; however, the percentage of those aged 50 years or older ranged from 34.5% (Taiwan) to 81.8% (South Korea). The minimum/maximum percentages of qualified OPT practitioner were 6.8% (Portugal) and 65.9% (South Korea), and the percentages of those who received OPT for the first time or for the first time in a long time ranged from 9.1% (South Korea) to 82.2% (Thailand). Those who underwent the session at an MOA affiliated institute or workshop accounted for more than 70% in each country, except for Japan (27.0%, Table 3).

**Table 3.** Representative Patients' Characteristics in Each Country

	Female gender	Aged ≥50 years	OPT practitioner	Never/seldom OPT experience	Therapy at an MOA institute or workshop
Japan	5506/7083 (77.7%)	5468/7158 (76.4%)	4587/7131 (64.3%)	2170/7045 (30.8%)	1869/6910 (27.0%)
The United States	888/1194 (74.4%)	880/1253 (70.2%)	496/1226 (40.5%)	699/1244 (56.2%)	1108/1207 (91.8%)
Thailand	905/1164 (77.7%)	587/1173 (50.0%)	215/1171 (18.4%)	963/1171 (82.2%)	1057/1123 (94.1%)
Chile/Peru	816/1030 (79.2%)	624/1032 (60.5%)	120/1018 (11.8%)	711/994 (71.5%)	967/1024 (94.4%)
Portugal	94/132 (71.2%)	77/134 (57.5%)	9/132 (6.8%)	105/130 (80.8%)	87/131 (66.4%)
Spain	83/105 (79.0%)	54/110 (49.1%)	18/106 (17.0%)	88/109 (80.7%)	109/113 (96.5%)
Argentina	85/104 (81.7%)	57/102 (55.9%)	50/104 (48.1%)	37/104 (35.6%)	75/105 (71.4%)
Mexico	61/79 (77.2%)	44/79 (55.7%)	30/77 (39.0%)	31/76 (40.8%)	80/81 (98.8%)
Brazil	42/52 (80.8%)	40/52 (57.7%)	25/51 (49.0%)	23/53 (43.4%)	48/56 (85.7%)
South Korea	29/44 (65.9%)	36/44 (81.8%)	29/44 (65.9%)	4/44 (9.1%)	40/44 (90.9%)
Taiwan	19/29 (65.5%)	10/29 (34.5%)	16/29 (55.2%)	11/28 (39.3%)	23/28 (82.1%)
Belgium/France	21/25 (84.0%)	20/25 (80.0%)	12/25 (48.0%)	7/25 (28.0%)	17/24 (70.8%)

a/b (c%) is expressed as the minimum or the maximum percentage in each characteristic.

OPT, Okada Purifying Therapy.

A total of 3110 investigators who were identified by the registration number on the record sheets administered a single session of OPT to 9954 participants (88.1%), whereas the investigator's registration number remained blank in the remaining 1349 record sheets (11.9%). Of the 3110 investigators, 2125 (68.3%) administered OPT to 1–2 participants, 661 (21.3%) administered it to 3–5 subjects, 199 (6.4%) to 6–9 subjects, and the remaining 125 (4.0%) contributed more, ranging between 10 and 130 subjects.

### Self-reported changes in overall symptoms

**FIG. 2.**
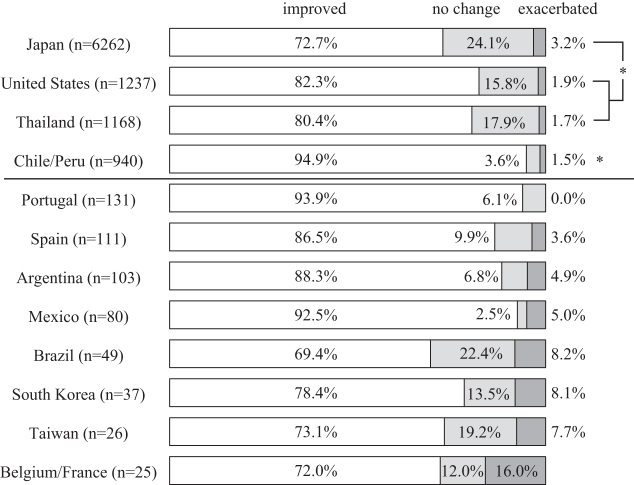
Self-reported changes in overall symptoms after a single session of Okada Purifying Therapy. *p < 0.0083 by the Mann–Whitney test between the participants from Japan, the United States, Thailand, and Chile/Peru.

Of the total 10,169 participants who met the inclusion criteria for analyses, 7885 (77.5%) self-reported an improvement of symptoms, 1999 (19.7%) described no change, and the remaining 285 (2.8%) experienced exacerbation of symptoms. Participants from Chile/Peru reported symptom improvement more often than those from Japan, the United States, and Thailand (*p* < 0.0083). Those from Japan included the lowest proportion of individuals who claimed symptom improvement (*p* < 0.0083). Spanish speaking countries and Portugal showed high improvement rates (Fig. 2).

The logistic regression analysis indicated that the following factors were independently associated with the symptom improvement: participants' country of residence, gender, reasons for participation, MQL-10 score, qualification as an OPT practitioner, previous OPT experience, location, and duration of the OPT administration (Table 4).

**Table 4.** Logistic Regression Analysis of the Factors Associated with an Improvement of Overall Symptoms (*n* = 7691)

	Imp. R	p-Value	Odds ratio (95% CI)
Country of residence			
Japan	72.3%		1
The United States	81.5%	<0.001	2.01 (1.60–2.54)
Thailand	79.9%	<0.001	2.32 (1.87–2.90)
Chile/Peru	95.0%	<0.001	8.31 (5.33–13.0)
Age		0.051	
Gender			
Male	66.6%		1
Female	78.3%	<0.001	1.66 (1.47–1.88)
Presence of illness		0.25	
Reasons for participation			
Health promotion	82.4%		1
Symptom relief	83.9%	0.039	1.21 (1.01–1.44)
Suggestion of others	66.5%	<0.001	0.64 (0.54–0.75)
MQL-10 score			
Low (≤25)	76.0%		1
Moderate (26–30)	77.2%	0.50	
High (≥31)	72.9%	0.049	0.87 (0.75–0.99)
OPT practitioner			
No	70.0%		1
Yes	80.7%	<0.001	1.37 (1.17–1.61)
Previous OPT experience			
Never/seldom	69.4%		1
Sometimes/regularly	80.3%	0.001	1.32 (1.12–1.55)
Location of OPT administration			
Participant's home	67.9%		1
MOA's institute/workshop	82.5%	<0.001	1.57 (1.33–1.84)
Investigator's house	72.6%	0.033	1.17 (1.01–1.35)
Duration of OPT administration			
30 min	72.1%		1
31–50 min	72.9%	0.002	1.23 (1.08–1.41)
≥51 min	83.2%	<0.001	1.78 (1.53–2.07)

Categorical values are shown as proportions (%). Reference category: no change or exacerbation in symptoms.

CI, confidence interval; Imp. R, improvement rate of symptoms in each factor; MQL-10, 10-item MOA quality-of-life questionnaire; OPT, Okada Purifying Therapy.

### Self-reported changes in physical pain

**FIG. 3.**
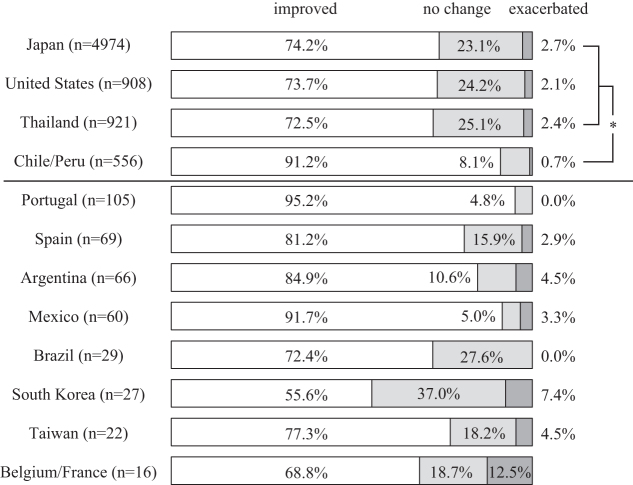
Self-reported changes in physical pain after a single session of Okada Purifying Therapy. *p < 0.0083 by the Mann–Whitney test between the participants from Japan, the United States, Thailand, and Chile/Peru.

Of the total 7753 participants who met the inclusion criteria for analyses, 5865 (75.6%) stated an improvement in their symptoms, the level remained unchanged for 1696 (21.9%), and 192 (2.5%) expressed symptom exacerbation. Participants from Chile/Peru reported symptom improvement more often than those from Japan, the United States, and Thailand (*p* < 0.0083). Spanish speaking countries and Portugal also showed high improvement rates (Fig. 3).

The logistic regression analysis indicated that the following factors were independently associated with the symptom improvement: participants' country of residence, gender, previous OPT experience, and duration of the OPT administration (Table 5).

**Table 5.** Logistic Regression Analysis of the Factors Associated with an Improvement of Physical Pain (*n* = 5926)

	Imp. R	p-Value	Odds ratio (95% CI)
Country of residence			
Japan	73.8%		1
The United States	73.9%	0.11	
Thailand	72.0%	0.016	1.31 (1.05–1.64)
Chile/Peru	89.4%	<0.001	3.78 (2.50–5.71)
Age		0.15	
Gender			
Male	71.5%		1
Female	75.1%	0.028	1.18 (1.02–1.36)
Presence of illness		0.40	
Reasons for participation		0.051	
MQL-10 score		0.10	
OPT practitioner		0.58	
Previous OPT experience			
Never/seldom	71.1%		1
Sometimes/regularly	76.5%	0.021	1.24 (1.03–1.49)
Location of OPT administration		0.76	
Duration of OPT administration			
30 min	70.8%		1
31–50 min	74.1%	0.011	1.21 (1.05–1.41)
≥51 min	78.8%	<0.001	1.51 (1.29–1.77)

Categorical values are shown as proportions (%). Reference category: no change or exacerbation in symptoms.

CI, confidence interval; Imp. R, improvement rate of symptoms in each factor; MQL-10, 10-item MOA quality-of-life questionnaire; OPT, Okada Purifying Therapy.

### Self-reported changes in anxiety/depression

**FIG. 4.**
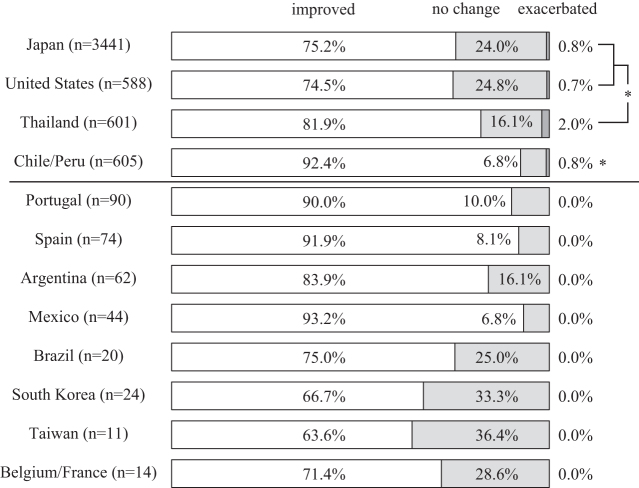
Self-reported changes in anxiety/depression after a single session of Okada Purifying Therapy. *p < 0.0083 by the Mann–Whitney test between the participants from Japan, the United States, Thailand, and Chile/Peru.

Of the total 5574 participants who met the inclusion criteria for analyses, 4368 (78.4%) reported an improvement in symptoms, 1159 (20.8%) described no change, and the remaining 47 (0.8%) experienced exacerbation of symptoms. Chile/Peru had the highest proportion of individuals who expressed symptom improvement among the four countries (*p* < 0.0083). Those from Thailand reported symptom improvement more often than those from Japan and the United States (*p* < 0.0083). Spanish speaking countries and Portugal also showed high improvement rates (Fig. 4).

The logistic regression analysis indicated that the participants' country of residence, location, and duration of the OPT administration were independently associated with the symptom improvement (Table 6).

**Table 6.** Logistic Regression Analysis of the Factors Associated with an Improvement of Anxiety/Depression (*n* = 4171)

	Imp. R	p-Value	Odds ratio (95% CI)
Country of residence			
Japan	75.4%		1
The United States	75.3%	0.59	
Thailand	81.9%	<0.001	1.73 (1.28–2.33)
Chile/Peru	91.2%	<0.001	3.73 (2.40–5.79)
Age		0.40	
Gender		0.066	
Presence of illness		0.77	
Reasons for participation		0.080	
MQL-10 score		0.59	
OPT practitioner		0.71	
Previous OPT experience		0.55	
Location of OPT administration			
Participant's home	72.2%		1
MOA's institute/workshop	80.3%	0.098	
Investigator's house	78.7%	0.007	1.32 (1.08–1.61)
Duration of OPT administration			
30 min	75.7%		1
31–50 min	75.5%	0.038	1.22 (1.01–1.46)
≥51 min	81.5%	<0.001	1.74 (1.42–2.12)

Categorical values are shown as proportions (%). Reference category: no change or exacerbation in symptoms.

CI, confidence interval; Imp. R, improvement rate of symptoms in each factor; MQL-10, 10-item MOA quality-of-life questionnaire; OPT, Okada Purifying Therapy.

### Self-reported changes in dizziness/palpitation

**FIG. 5.**
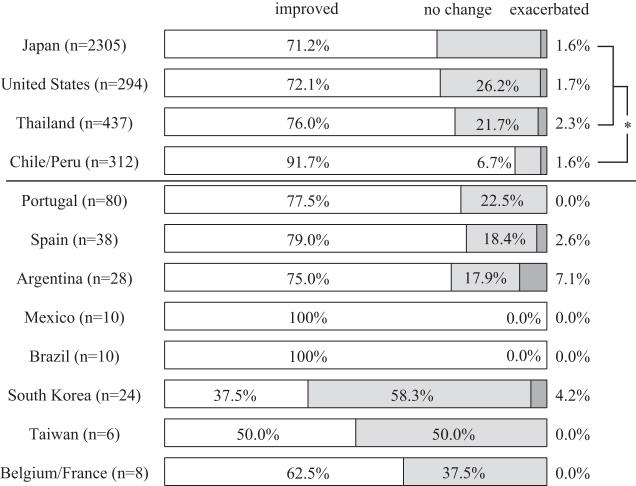
Self-reported changes in dizziness/palpitation after a single session of Okada Purifying Therapy. *p < 0.0083 by the Mann–Whitney test between the participants from Japan, the United States, Thailand, and Chile/Peru.

Of the total 3552 participants who met the inclusion criteria for the analyses, 2622 (73.8%) described an improvement of symptoms, 869 (24.5%) reported no change, and the remaining 61 (1.7%) expressed exacerbation of symptoms. Participants from Chile/Peru reported symptom improvement more often than those from Japan, the United States, and Thailand (*p* < 0.0083). Spanish/Portuguese speaking countries also showed high improvement rates (Fig. 5).

The logistic regression analysis indicated that participants' country of residence, location, and duration of the OPT administration were independently associated with symptom improvement (Table 7).

**Table 7.** Logistic Regression Analysis of the Factors Associated with an Improvement of Dizziness/Palpitation (*n* = 2704)

	Imp. R	p-Value	Odds ratio (95% CI)
Country of residence			
Japan	71.8%		1
The United States	71.4%	0.42	
Thailand	77.0%	0.001	1.79 (1.29–2.51)
Chile/Peru	90.6%	<0.001	4.29 (2.44–7.57)
Age		0.98	
Gender		0.26	
Presence of illness		0.87	
Reasons for participation		0.32	
MQL-10 score		0.66	
OPT practitioner		0.43	
Previous OPT experience		0.11	
Location of OPT administration			
Participant's home	67.7%		1
MOA's institute/workshop	75.3%	0.34	
Investigator's house	78.3%	<0.001	1.68 (1.32–2.15)
Duration of OPT administration			
30 min	72.4%		1
31–50 min	71.3%	0.55	
≥51 min	77.2%	0.002	1.44 (1.14–1.82)

Categorical values are shown as proportions (%). Reference category: no change or exacerbation in symptoms.

CI, confidence interval; Imp. R, improvement rate of symptoms in each factor; MQL-10, 10-item MOA quality-of-life questionnaire; OPT, Okada Purifying Therapy.

### Adverse events of OPT

Among the 228 participants who reported exacerbation of symptoms after the intervention and what they had done to relieve their symptoms, 178 (78.1%) waited it out, 46 (20.1%) received further OPT session, 2 (0.9%) took medications, and the remaining 2 (0.9%) consulted a doctor. Of the 169 individuals who described the duration of symptom exacerbation, 121 (71.6%) recovered in a few hours, and 19 (11.2%) recovered in 24 h. The exacerbation persisted for more than 24 h in the remaining 29 (17.2%). None of them needed emergency medical treatment.

### Results of participants from Japan

Of the participants from Japan who met the inclusion criteria for analyses, duration of the OPT administration was the consistent factor associated with the changes in physical pain, anxiety/depression, and dizziness/palpitation. Location of the session was also the independent factor associated with changes in anxiety/depression and dizziness/palpitation. Previous OPT experience was associated with changes in physical pain. Participants' gender was related to the changes in anxiety/depression (Table 8).

**Table 8.** Logistic Regression Analyses of the Factors Associated with an Improvement of Symptoms in Participants from Japan

	Physical pain Odds ratio (95% CI)	Anxiety/depression Odds ratio (95% CI)	Dizziness/palpitation Odds ratio (95% CI)
Age			
Gender			
Male		1	
Female		1.24 (1.01–1.51)	
Presence of illness			
Reasons for participation			
MQL-10 score			
OPT practitioner			
Previous OPT experience			
Never/seldom	1		
Sometimes/regularly	1.29 (1.06–1.59)		
Location of OPT administration			
Participant's home		1	1
MOA's institute/workshop			
Investigator's house		1.35 (1.10–1.65)	1.67 (1.31–2.14)
Duration of OPT administration			
30 min	1	1	1
31–50 min	1.19 (1.01–1.41)	1.30 (1.06–1.59)	
≥51 min	1.49 (1.25–1.78)	1.70 (1.37–2.11)	1.48 (1.15–1.90)

Categorical values are shown as proportions (%). Reference category: no change or exacerbation in symptoms.

MQL-10, 10-item MOA quality-of-life questionnaire; OPT, Okada Purifying Therapy.

### Influence of qualification as an OPT practitioner on symptom improvement

After receiving a single session of OPT at an MOA-affiliated institute or workshop, the improvement rates of overall symptoms differed between those with never/seldom OPT experience and qualified OPT practitioners in the individuals from Japan (66.2%/83.5%), the United States (75.7%/90.9%), and Thailand (76.8%/96.4%), respectively (*p* < 0.05), but not in those from Chile/Peru (94.1%/98.8%). As for other symptoms, the improvement rates did not differ between the two groups, except for physical pain in those from Thailand (70.2%/84.4%, *p* < 0.05) and dizziness/palpitation in the subjects from the United States (66.4%/82.3%, *p* < 0.05) (Table 9).

**Table 9.** Improvement Rates of Symptoms After Receiving Okada Purifying Therapy at an MOA Affiliated Institute or Workshop

	OPT practitioner	Overall symptoms	Physical pain	Anxiety/depression	Dizziness/palpitation
Japan	No	157/237 (66.2%)	130/170 (76.5%)	91/119 (76.5%)	52/81 (64.2%)
	Yes	919/1101 (83.5%)^*^	715/967 (73.9%)	473/616 (76.8%)	330/462 (71.4%)
United States	No	412/544 (75.7%)	262/364 (72.0%)	216/286 (75.5%)	91/137 (66.4%)
	Yes	320/352 (90.9%)^*^	216/284 (76.1%)	101/133 (75.9%)	65/79 (82.3%)^*^
Thailand	No	638/831 (76.8%)	455/648 (70.2%)	341/423 (80.6%)	250/323 (77.4%)
	Yes	106/110 (96.4%)^*^	81/96 (84.4%)^*^	55/63 (87.3%)	33/44 (75.0%)
Chile/Peru	No	524/557 (94.1%)	285/317 (89.9%)	332/357 (93.0%)	168/184 (91.3%)
	Yes	80/81 (98.8%)	51/56 (91.1%)	49/56 (87.5%)	33/34 (97.1%)

a/b (c%)^*^ indicates the *p*-value between the comparison of the improvement rate in “No” OPT practitioners (those with never/seldom OPT experience) and the rate in “Yes” OPT practitioners (qualified OPT practitioners) is less than 0.05 by Mann–Whitney test.

OPT, Okada Purifying Therapy.

## Discussion

This study adopted the convenience sampling method, which inevitably led to the recruitment of subject groups biased in favor of people sympathetic to or skilled in OPT. Moreover, a large proportion of treatments occurred in an authorized location where OPT was promoted. In addition, there were large discrepancies in the participants' characteristics between countries. While these shortcomings remain, nonetheless, this study is presumably the first international survey conducted to examine ethnic/cultural differences in the outcomes of biofield therapy.

Past studies have reported on numerous factors associated with the use of biofield therapy, such as the recipient's ethnicity, gender, socioeconomic status, and/or health condition.^10,11,31–34,39,40^ In terms of factors associated with improved outcomes, the research team previously reported that female gender, positive reasons for receiving the therapy, an authorized location, and a longer therapy were independently associated with improvement of various symptoms after a single OPT session in individuals from Japan.^24^ This is in line with study by Kristoffersen et al., which indicated a higher frequency of self-reports from female than male clients on improvement of various symptoms after receiving energy healing.^10^

The present study revealed that participants from Chile/Peru self-reported the improvement in overall symptoms, physical pain, anxiety/depression, and dizziness/palpitation more often than those from Japan, the United States, and Thailand. Participants from Japan, on the contrary, consistently indicated the lowest improvement rate for each of the different symptoms. Even after adjusting for other demographic factors, participant's country of residence still remained the most significant factor associated with the changes in symptoms.

Interestingly, those from Spanish/Portuguese speaking countries almost always showed higher improvement rates in various symptoms compared to other countries. Haack et al. mentioned that Latin America is a culturally rich region of the world with diverse perspectives on health.^41^ People living in those countries are often more holistic and spiritual and pay greater attention to psychologic, social, emotional, and spiritual aspects of disorder based on their cultural beliefs.^38,41,42^ In contrast, population in Japan usually rely on Western medicine, which is easy to access and paid by the national insurance. Laying-on-of-hands is neither a major Japanese traditional healing practice nor a common religious ritual, and thus many of these subjects may have been skeptical toward the effectiveness of OPT from the beginning.

On the contrary, Thailand is a notable Buddhist country, and the United States is a multiethnic country that comprises immigrants who are linked to the culture of origin with discrepancy in the degree of acculturation. For instance, African Americans reportedly have more spiritual mindset than other ethnic groups.^43^ Furthermore, Bair et al. described that white and Chinese American women often used spiritual therapies when they suffered psychologic symptoms, whereas Japanese American women did not.^44^

Participants who held strong ethnic identities and/or those who had spiritual mindset might call their own indigenous practices to mind during the OPT session. Alternatively, Japanese descendants may have had a positive or negative interpretation of OPT, which was often introduced in the Japanese community. Various proportions of such individuals in the population may have been represented in the study, which resulted in differences of changes in symptoms between countries.

Perception of symptoms may also differ based on ethnicity. Hispanic and/or people of African descent were reportedly more likely to suffer from physical pain,^45,46^ higher pain intensity,^46^ and antenatal depression^47,48^ than non-Hispanic white. Aufiero et al. described from their experimental study that Latino Americans and women reported greater pain with a standardized pain stimulus compared to Caucasian Americans and men.^49^ Those suffering from marked symptoms may have been relieved to some extent more often than those with mild symptoms.

In addition, ethnicity and/or culture may moderate placebo response. Walach and Jonas defined placebo response as the effect that is due to the meaning of a therapeutic intervention for a particular patient and context.^35^ They additionally described that the meaning arises from the interaction between the external environment and the internal conditions of persons, their history, social circumstances, individual predilections, and their expectations. In the present study, several factors, including location and duration of the OPT administration, and reasons for participation were likely to be associated with placebo response. Race and ethnicity may also influence the quality of provider–client interactions.^36,37^ The mere fact of being observed may have induced improvement in symptoms for some participants (positive Hawthorne effect),^50–52^ on the contrary, some may have overexpressed symptoms, for instance, as a means to express the desire to be taken seriously (negative Hawthorne effect).^52^ Leurent et al. described the presence of Hawthorne effect in health professionals, who changed their practices when they were being observed.^53^ Investigators' expectations (Pygmalion effect) may also have affected participants' judgment on the effectiveness of OPT.^54,55^ These factors may have increased the outcome differences among participants with ethnic/cultural differences.

Comparison between the present study and the team's previous study^24^ revealed that the improvement rates of physical pain, anxiety/depression, and dizziness/palpitation in the present study were consistently higher than in the previous study; 74.2% versus 69.7%, 75.2% versus 71.2%, and 71.2% versus 67.5%, respectively. A longer OPT administration was consistently associated with symptom improvement in both studies. The investigators who provided the therapy may have continued the treatment until the participants felt better. Alternatively, the participants may have reported positive changes due to psychologic satisfaction from longer treatments.^24^ Location of the administration and participants' gender remained the factors related to the improvement of different symptoms.

In the present study, qualified OPT practitioners did not always report their symptom changes more positively than did those with never/seldom OPT experience, regardless of their country of residence. Reece et al. reported that nonpractitioners of Johrei healing experienced a greater effect after the session than practitioners.^16^ Sufficient information may provide both advantages and disadvantages to the effectiveness of various therapies. Individuals with qualification as a practitioner and/or sufficient previous experience may have compared the particular experience of therapy received during the study with their past impressive experience, drawing a critical evaluation.

The exacerbation rates of symptoms after a single OPT administration varied in the range of 0.7% and 2.5% in the team's previous study.^24^ The present study revealed that 0.8% to 2.8% of the participants experienced symptom exacerbation. None of them needed emergency medical treatment, in which case OPT may be a useful complementary method for individuals in various situations, including for those seeking whole-person health, those wishing to recover from various symptoms, and for those receiving end-of-life care.

There were several limitations in the study design. First, the survey was not experimental as it used a convenience sample without an appropriate control or comparison group. As mentioned earlier, this study included a high percentage of elderly and/or female participants, and those having a qualification of OPT practitioner. In addition, OPT was frequently administered in an authorized location. All of these factors may have created a bias. The present study reveals neither the effectiveness of OPT in the general population nor of effectiveness of general biofield therapy.

Second, it may have been difficult for recipients to reveal their honest evaluations in the situation that their responses were presented to practitioners with whom they worked. Practitioners may have had recipients self-report a better outcome intentionally or unintentionally.

Third, some data may have not been recorded correctly during the data collection and/or during data entry for analyses. The possibility of potential cheating, such as changing of scores on the records by the practitioners, also cannot be denied.

Fourth, the information sheet and the questionnaires may not have been properly translated into each respective language. Moreover, some symptoms such as anxiety, dizziness, and palpitation may have different meanings in different languages, which influence the outcomes among those with ethnic/cultural differences.

Fifth, the factors that were assessed in the questionnaire were not exhaustive, and other factors may have contributed to the outcomes. Additional studies with rigorous study protocols are warranted to further investigate the effectiveness of OPT.

## Conclusions

In those who were often sympathetic to OPT and/or in an authorized location, a single session of the therapy lasting 30 min or longer administered from volunteer practitioners was effective and safe across countries with ethnic/cultural differences. The improvement rates were consistently higher, among participants from Chile/Peru than those from Japan, the United States, and Thailand, and among those who had received a longer therapy. Spanish/Portuguese speaking countries almost always showed high improvement rates, conversely, Japan showed a lower rate in each symptom. Participants' gender, reasons for participation, previous experience, and location of receiving the session were also associated with the improvement of different symptoms.

Most of the participants who expressed exacerbation of symptoms recovered in a few hours. None of them needed emergency medical treatment.
